# Using Linkage Maps as a Tool To Determine Patterns of Chromosome Synteny in the Genus *Salvelinus*

**DOI:** 10.1534/g3.117.300317

**Published:** 2017-09-29

**Authors:** Matthew C. Hale, Garrett J. McKinney, Courtney L. Bell, Krista M. Nichols

**Affiliations:** * Department of Biological Sciences, Purdue University, West Lafayette, Indiana 47907; † Department of Biology, Texas Christian University, Fort Worth, Texas 76129; ‡ School of Aquatic and Fisheries Sciences, University of Washington, Seattle, Washington 98105; § Conservation Biology Division, Northwest Fisheries Science Center, National Marine Fisheries Service, National Oceanic and Atmospheric Administration, Seattle, Washington 98115

**Keywords:** linkage mapping, SNPs, salmonids, synteny, recombination

## Abstract

Next generation sequencing techniques have revolutionized the collection of genome and transcriptome data from nonmodel organisms. This manuscript details the application of restriction site-associated DNA sequencing (RADseq) to generate a marker-dense genetic map for Brook Trout (*Salvelinus fontinalis*). The consensus map was constructed from three full-sib families totaling 176 F_1_ individuals. The map consisted of 42 linkage groups with a total female map size of 2502.5 cM, and a total male map size of 1863.8 cM. Synteny was confirmed with Atlantic Salmon for 38 linkage groups, with Rainbow Trout for 37 linkage groups, Arctic Char for 36 linkage groups, and with a previously published Brook Trout linkage map for 39 linkage groups. Comparative mapping confirmed the presence of 8 metacentric and 34 acrocentric chromosomes in Brook Trout. Six metacentric chromosomes seem to be conserved with Arctic Char suggesting there have been at least two species-specific fusion and fission events within the genus *Salvelinus*. In addition, the sex marker (*sdY*; sexually dimorphic on the Y chromosome) was mapped to Brook Trout BC35, which is homologous with Atlantic Salmon Ssa09qa, Rainbow Trout Omy25, and Arctic Char AC04q. Ultimately, this linkage map will be a useful resource for studies on the genome organization of *Salvelinus*, and facilitates comparisons of the *Salvelinus* genome with *Salmo* and *Oncorhynchus*.

Genetic linkage maps are useful tools in evolutionary genetics for the discovery of Quantitative Trait Loci (QTL), comparative genomics, and in anchoring sequences to specific chromosomal regions. Their use in comparative genomics between nonmodel and model organisms is important as linkage maps can facilitate the identification of candidate genes for traits of interest (Gross *et al.* 2008; Sarropoulou and Fernandes 2011). Moreover, advancements in sequencing technology have revolutionized the collection of genetic data on nonmodel organisms allowing linkage maps to be quickly constructed in species with limited genetic data (*e.g.*, Sutherland *et al.* 2016). Despite their uses and the increased ease of their construction, linkage maps in wild species are still scarce (Ellegren 2013).

Salmonids are of interest from an evolutionary and economical perspective (Davidson *et al.* 2010), and linkage maps are important resources that facilitate the location of genes connected to the development of traits of interest. To that end, there are marker-dense linkage maps available for Atlantic Salmon (*Salmo salar*; Danzmann *et al.* 2008; Moen *et al.* 2008; Lien *et al.* 2011), Rainbow Trout (*Oncorhynchus mykiss*; Sakamoto *et al.* 2000; Nichols *et al.* 2003; Miller *et al.* 2012; Palti *et al.* 2015), Sockeye Salmon (*O. nerka*; Everett *et al.* 2012; Larson *et al.* 2016; Everett and Seeb 2014), Coho Salmon (*O. kisutch*; Kodama *et al.* 2014), Chum Salmon (*O. keta*; Waples *et al.* 2016, Chinook Salmon (*O. tshawytscha*; Brieuc *et al.* 2014; Everett and Seeb 2014; McKinney *et al.* 2016), Pink Salmon (*O. gorbuscha*; Limborg *et al.* 2014), Arctic Char (*Salvelinus alpinus*; Nugent *et al.* 2017), and Brook Trout (*S. fontinalis*; Sutherland *et al.* 2016). Many of these maps have used Genotype By Sequencing (GBS) approaches and consist of thousands of SNPs distributed throughout the genome. In addition, there are genome sequences available for both *O. mykiss* (Berthelot *et al.* 2014) and *S. salar* (Lien *et al.* 2016), as well as numerous EST and genome scaffold resources for both species (Palti *et al.* 2011, 2014; Davidson *et al.* 2010).

Despite the large amount of available genetic data, salmonid genomics faces several challenges, not least an ancestral salmonid-specific (4R) genome duplication that occurred early in salmonid evolution (Allendorf and Thorgaard 1984). Although salmonid genomes are in the process of rediplodizing, a portion of the genome is still undifferentiated and can exhibit tetrasomic inheritance (∼10% of the Atlantic Salmon genome; Lien *et al.* 2011, 2016) Failure to identify duplicates (paralogous sequence variants) is problematic as it is difficult to infer gene dosage (and meiotic phase) for paralogs using GBS approaches; this can result in incorrect estimates of recombination (Waples *et al.* 2016).

Brook Trout (*S. fontinalis*) is a species of salmonid native to the northern United States and Canada. Although research on Brook Trout has been less intensive than on other salmonids, recent studies have described variation in several evolutionary traits of interest such as morphology, size, age of sexual maturation, and water temperature tolerance (*e.g.*, Varian and Nichols 2010; McKinney *et al.* 2014; McDermid *et al.* 2012; Serfas *et al.* 2012; Kazyak *et al.* 2013). Many of these traits have been shown to have a genetic basis in other salmonids, including development rate in Rainbow Trout (Sundin *et al.* 2005; Nichols *et al.* 2007; Miller *et al.* 2012), age at maturation in Atlantic Salmon (Barson *et al.* 2015), migration run timing in Chinook Salmon and Rainbow Trout (Hess *et al.* 2016), and temperature tolerance in Chinook Salmon (Everett and Seeb 2014) and Rainbow Trout (Narum *et al.* 2013). However, it is unknown whether the genetic architectures for these traits are conserved between these species and Brook Trout. Having multiple linkage maps for Brook Trout would allow comparisons to be made between the organization of the genome between different populations, a necessary first step in determining the genetic architecture of traits of interest.

The *Salvelinus* karyotype consists of ∼80 chromosomes, 100 chromosome arms, and more acrocentric than metacentric chromosomes (the salmonid “Type A” karyotype; Phillips and Rab 2001). *Oncorhynchus* and *Salmo* have a “Type B” karyotype, which is characterized by having a diploid number of chromosomes close to 60, ∼100 chromosome arms, and more metacentric than acrocentric chromosomes (Hartley 1987; Phillips and Rab 2001). Until recently, the only available linkage maps for *Salvelinus* were constructed from <350 microsatellite markers (Woram *et al.* 2004; Timusk *et al.* 2011; Sauvage *et al.* 2012). However, Sutherland *et al.* (2016) mapped <4000 SNP markers in one family of Brook Trout produced by crossing one wild anadromous female from Laval River, Quebec with a male from a domestic population (Sutherland *et al.* 2016). Although this increases the amount of genomic information for *Salvelinus*, standing genetic variation is population-specific. Loci that are fixed in one population can be variable in another population. Therefore, constructing linkage maps using different populations of the same species will increase the addition of ordered polymorphic markers. In addition, the karyotypes of several salmonids vary between different populations of the same species [*e.g.*, Arctic Char (Moghadam *et al.* 2007), Rainbow Trout (Thorgaard 1983), and Atlantic Salmon (Brenna-Hansen *et al.* 2012)]. Linkage maps are useful tools in comparative genomics, as they allow synteny to be compared between different populations of the same species. Therefore, the goals of this manuscript are twofold: (1) to produce a linkage map for Brook Trout using RADseq methods and (2) to compare the linkage map to linkage maps of Brook Trout (Sutherland *et al.* 2016), Arctic Char (Nugent *et al.* 2017), Rainbow Trout (Miller *et al.* 2012; Palti *et al.* 2015), and Atlantic Salmon (Lien *et al.* 2016) using the Atlantic Salmon genome as a reference and the program MapComp (Sutherland *et al.* 2016). Thus, we aim to increase the understanding the organization of the *Salvelinus* genome, both with respect to comparisons between *Salvelinus* and other salmonid genera, and between different species of *Salvelinus*.

## Materials and Methods

### Sampling and sequencing

Three F_1_ families were generated by crossing three adult male Brook Trout from Siskiwit River, MI with three adult females from Tobin Harbor, MI. Both populations spawn on and around Isle Royale, MI. Young fish then spend a period of time (typically several years) feeding in Lake Superior before returning to natal spawning grounds. Both populations have been used in restocking efforts in Lake Superior since the 1990s (Schreiner *et al.* 2008). The Tobin Harbor strain represents a lacustrine coaster population (*i.e.*, spend all their life in Lake Superior), and the Siskiwit River population was founded from adfluvial fish (*i.e.*, spawn in tributaries to Lake Superior on Isle Royale and then migrate to Lake Superior). The two strains are genetically distinct from each other (*F*_st_ = 0.13; Cooper *et al.* 2010; Stott *et al.* 2010). The Siskiwit River population has been used for heritability studies of phenotypes connected with migration (Varian and Nichols 2010; McKinney *et al.* 2014). The Tobin Harbor population has been used to study the ecological differences between coaster and stream living (fluvial) Trout (Huckins and Baker 2008). Crosses were made by applying light pressure on the abdomen and collecting gametes. Gametes were stored for <24 hr at 4° before fertilization. Fertilized embryos were shipped to the aquaculture facility at Purdue University, where all embryos were incubated and reared. Samples were kept in oxygenated water maintained at 8° and kept in constant darkness. A total of 176 F_1_ samples were generated: 52 from family 1, 54 from family 2, and 70 from family 3. After 55 d post fertilization samples were killed with a lethal dose of MS-222 (Argent Chemicals, Redmond, WA) and placed in 100% ethanol. DNA was extracted from tail tissue via a modified Phenol–Chloroform extraction protocol described in Hecht *et al.* (2012). DNA quality was assessed quantitatively using a Qubit (ThermoFisher, TX), and qualitatively by running 3 μl on a 1.5% agarose gel stained with ethidium bromide and viewed under UV light. Illumina RADseq was performed on all 182 (parents and F_1_) samples and RAD libraries were prepared following Miller *et al.* (2012). GBS loci identified by *Sbf*I-linked Illumina sequencing has been used for SNP discovery in multiple salmonid linkage maps (Everett *et al.* 2012; Miller *et al.* 2012; Brieuc *et al.* 2014; Waples *et al.* 2016) and we employed a similar methodology using *Sbf*I. Thirty-two samples were pooled on a lane, and six lanes of 100 bp single-end sequencing were conducted on an Illumina HiSequation 2000.

### SNP discovery

Raw sequences were quality filtered (minimum Q score of 20) and trimmed (3′ end) to 76 bp using the program using the process RADtags script in STACKS (Catchen *et al.* 2011). Trimmed sequences from the six parents were individually aligned in ustacks using the “bounded” genotyping model (low = 0.001, high = 0.01) with a minimum stack depth of 10 reads. These stacks were then used to create a catalog of loci in cstacks with a maximum number of two mismatches allowed between any candidate locus. All loci within this database were then compared against themselves to remove repeat sequences using Bowtie 2 (v 2.3.0; Langmead and Salzberg 2012), allowing up to two mismatches. Any locus that aligned to another locus in the catalog was removed from the database. Alignments for each parent and F_1_ sample were then performed using sstacks with default settings. Genotypes were calculated using the genotypes package in STACKs (default parameters), and SNPs scored in <80% of F_1_ samples removed. These filtering criteria produced a total of 12,961 candidate SNPs.

### Linkage mapping

Linkage maps were constructed using Lep-MAP v 2.0 (Rastas *et al.* 2013). Separate sex-specific maps of female segregating and male segregating loci were constructed because of the pronounced heterochiasmy exhibited by salmonids (Sakamoto *et al.* 2000). Pairwise estimates of linkage were carried out for all 12,961 candidate loci. First, the *SeperateChromosome* command was run with a minimum LOD score of 12, a maximum recombination fraction of 0.4, and a minimum number of markers per linkage group of 10. Any marker that showed evidence of segregation distortion (χ^2^ test *P* < 0.001) was removed. Unmapped markers were then rerun against the threshold map using the command *JoinSingles*, with a minimum LOD score of 5, a minimum LOD difference of 3, a maximum recombination fraction of 0.4, and Mendelian inheritance (χ^2^ test of segregation distortion *P* > 0.001). Markers were ordered using the *OrderMarkers* command using default parameters. This command rearranges the order of the markers on a linkage group and reports the “best” order (lowest LOD likelihood). Linkage groups were drawn using the program MAPCHARTv2.1 (Voorrips 2002).

### Synteny with other salmonids

To determine chromosomal organization with Brook Trout and other salmonid linkage maps we used a comparative approach using the program MapComp. This program compared the markers placed on the linkage map reported herein to the Brook Trout linkage map reported in Sutherland *et al.* (2016), the Arctic Char linkage map reported in Nugent *et al.* (2017), the Atlantic Salmon linkage map reported in Lien *et al.* (2016), and the Rainbow Trout linkage map reported in Miller *et al.* (2012). MapComp compares markers from different linkage mapping studies using their position on a related genome sequence [mapped sequences are aligned to a reference genome using BWA with default parameters (Li and Durbin 2009)]. RADseq loci were mapped to the reference genome if there was a single alignment and a MAPQ score > 10. The Atlantic Salmon genome was used as a reference genome for MapComp for all comparisons because it is more complete (*i.e.*, fewer gaps and unincorporated sequence) than the Rainbow Trout genome. Synteny between linkage groups was only inferred if >5 RADseq loci matched a specific linkage group.

### Genotyping and mapping sdY

The parents of all three mapping crosses were used as positive controls to validate whether *sdY* could be used to accurately determine sex in the F_1_ samples. PCR conditions followed those reported in Yano *et al.* (2013) using primers E2S1 and E2AS2. Reactions consisted of 0.1 mM of each primer, 5 μl of 2× Go-Taq PCR buffer (Promega), 50 ng of template DNA, and nanopure water to 10 μl. The presence of male-specific amplification was confirmed by running PCR products on a 1.5% agarose gel stained with GelRed (Biotium) and viewed under UV light. There was no incidence of misassignment between *sdY* and biological sex for any of the parents, therefore all F_1_ individuals were genotyped using the methods described above. PCR amplification for 10 candidate males and 10 candidate females was repeated twice to determine accurate assignment of sex. In no incidence was there a mismatch. *sdY* was added to the mapping dataset by scoring females as homozygotes and males as heterozygotes.

### Data availability

Supplemental Material, File S1 contains the input file for Lep-MAP showing genotypes for all three families for the 1990 mapped markers. File S2 contains the consensus sequence, marker ID, female map position, male map position, and linkage group for all mapped markers. File S3 shows the position of mapped markers in the Brook Trout linkage map. File S4 shows Oxford plots comparing the Brook Trout linkage map to (A) the Rainbow Trout linkage map, (B) The Atlantic Salmon linkage map, (C) The Brook Trout linkage map published in Sutherland *et al.* (2016), and (D) The Arctic Char linkage map. All Oxford plots were drawn by pairing mapped markers through the Atlantic Salmon genome. All RADseq data are uploaded in Data Dryad (doi: 10.5061/dryad.75mt7).

## Results

### Sequencing

Illumina RADseq produced a total of 427,823,712 quality-filtered sequences for the F1 samples and 39,938,749 quality filtered reads for the parents. The number of quality filtered reads varied from 780,935 to 12,936,817 for the F1 individuals (average number of QF reads = 3,145,762) and from 1,755,890 to 16,643,600 for the parents (average number of QF reads = 6,656,458).

### Linkage mapping and placement of the sex marker

**Figure 1:**
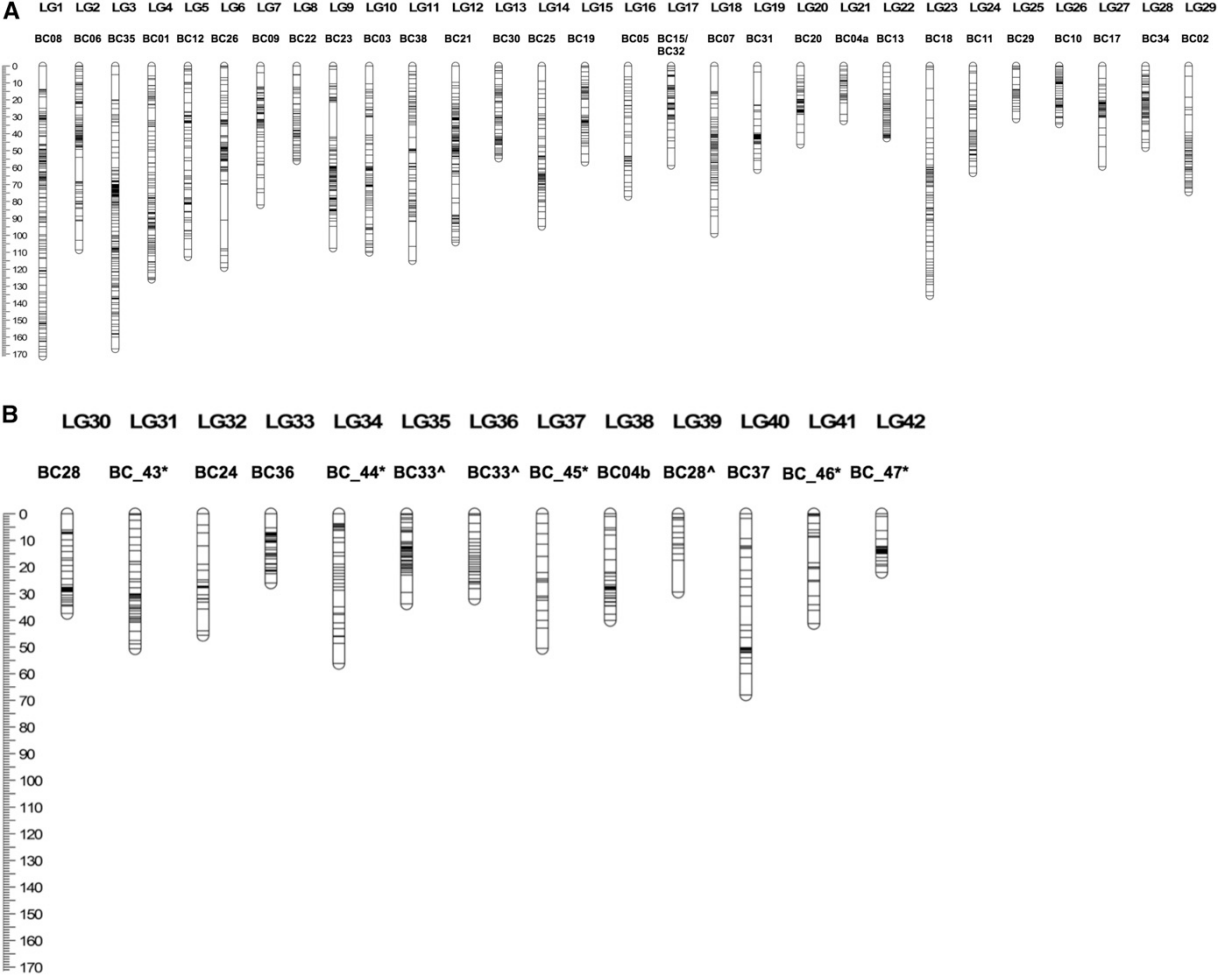
Sex-averaged Brook Trout linkage map. The bars on the linkage groups (LGs) represent mapped restriction site-associated DNA sequencing (RADseq) loci. Lengths are calculated in Kosambi cM. Two names per LG are given: The top name refers to the linkage map herein (allowing comparison with File S1, File S2, and File S3), whereas the bottom name allows comparison with Sutherland *et al.* 2016. ^ indicates that two linkage groups were aligned to the same linkage group in Sutherland *et al.* 2016 and * indicates homology inferred by a small number of markers.

A total of 12,961 unique RAD loci were discovered in three F1 families of Brook Trout. The final linkage map consisted of 1990 markers located on 42 linkage groups (genotypes provided in File S1, sequence and position of RAD loci provided in File S2). The number of mapped markers varied per family (1295 for family 1, 1923 for family 2, and 905 for family 3), of which 701 loci were shared between all three families, 728 were shared between two of the three families, and 561 loci were specific to a family. Linkage groups built from female informative meioses ranged from 0 to 185.1 cM with a total map size of 2502.5 cM. The male map totaled 1863.8 cM with individual linkage groups ranging in size from 0 to 112.9 cM (see Table 1 for summary statistics on linkage map and Figure 1 for the complete sex averaged map). These 42 linkage groups likely correspond to the 42 chromosomes described by previous linkage mapping studies in *Salvelinus*. The sex marker was place on BC35 in an area of low recombination with 26 other markers (File S3).

**Table 1:** Summary of the 42 linkage groups of Brook Trout with the number of markers and the average spacing of markers

LG	Size of LG in cM (Female Map)	Size of LG in cM (Male Map)	Number of Markers in LG	Average Spacing of Markers in cM (Females)	Average Spacing of Markers in cM (Males)
BC01	97.09	17.89	94	1.03	0.19
BC02	30.58	21.26	21	1.46	1.01
BC03	28.25	54.06	56	0.5	0.97
BC04a	17.17	52.07	39	0.44	1.34
BC04b	66.01	0.28	18	3.67	0.02
BC05	111.71	31.76	71	1.57	0.45
BC06	113.04	34.17	72	1.57	0.47
BC07	185.13	17.31	55	3.37	0.31
BC08	127.03	88.55	123	1.03	0.72
BC09	44.68	67.03	75	0.6	0.89
BC10	25.02	77.47	31	0.81	2.5
BC11	36.85	12.1	29	1.27	0.42
BC12	90.24	111.02	80	1.13	1.39
BC13	46.38	56.05	44	1.05	1.27
BC17	50.52	76.66	38	1.33	2.02
BC18	115.43	23.13	42	2.75	0.55
BC19	49.45	20.61	61	0.81	0.34
BC20	37.97	14.43	43	0.88	0.34
BC21	31.38	45.53	54	0.58	0.84
BC22	75.5	68.84	61	1.24	1.13
BC23	70.42	66.08	72	0.98	0.92
BC24	22.59	22.61	35	0.65	0.65
BC25	104.52	18.33	52	2.01	0.35
BC26	10.1	103.4	77	0.13	1.34
BC28	44.71	21.85	29	1.54	0.75
BC29	130.16	84.03	33	3.94	2.55
BC30	160.02	85.03	73	2.19	1.16
BC31	23.46	49.78	42	0.56	1.19
BC31	59.64	16.53	33	1.81	0.5
BC32	86.72	20.181	68	1.28	0.3
BC33	23.06	0	20	1.15	0
BC34	32.45	8.12	30	1.08	0.27
BC35	79.15	70.12	95	0.83	0.74
BC36	60.3	5.4	26	2.32	0.21
BC37	58,86	26.57	29	2.03	0.92
BC38	30.18	64.7	50	0.6	1.29
BC41	63.3	7.66	23	2.75	0.33
BC_43*	38.14	104.86	21	1.82	4.99
BC_44*	40.72	7.41	19	2.14	0.39
BC_45*	34.09	112.94	28	1.22	4.03
BC_46*	0	11.97	16	0	0.75
BC_47*	9.363	65.98	12	0.78	5.5

LG, linkage group.

### Comparisons within Salvelinus

MapComp was able to determine homology between the linkage map herein and in Sutherland *et al.* (2016) for all but three Brook Trout chromosomes (BC27, BC37, and BC42). These missing chromosomes likely reflect the small number of mapped markers on these chromosomes rather than differences in the karyotype between the two populations. In addition, five linkage groups in the study herein could not be matched to the Arctic Char linkage map in Sutherland *et al.* (2016), nor could they be placed (accurately) on the Atlantic Salmon genome. Comparisons with Sutherland *et al.* (2016) confirmed the presence of eight metacentric chromosomes (BC01–BC08) and 36 acrocentric chromosomes (BC09–BC42). MapComp determined homology for 35 Arctic Char linkage groups with Brook Trout, with AC09, AC34, and AC36 failing to produce homology with any Brook Trout linkage group (Nugent *et al.* 2017: Table 2). Three Arctic Char chromosome arms (AC01q, AC04q, and AC10) each matched two Brook Trout chromosome arms (BC03 and BC42, BC15 and BC35, and BC06 and BC28, respectively), suggesting separate chromosome rearrangement events after Arctic Char split from Brook Trout (discussed below). See Oxford plots showing synteny in File S4.

**Table 2:** All orthologous relationships between Brook Trout, Arctic Char, Atlantic Salmon, and Rainbow Trout as determined by MapComp using the Atlantic Salmon genome as a reference

Brook Trout Linkage Group (This Study)	Brook Trout Linkage Group (Sutherland *et al.* 2016)	Arctic Char Linkage Group (Nugent *et al.* 2017)	Atlantic Salmon Chromosome	Rainbow Trout Chromosome
**4**	**BC01**	AC18p	Ssa19qb	Omy16p
**4**	**BC01**	AC18	Ssa01qa	Omy23
**29**	**BC02**	AC03p	Ssa07p	Omy21p
**29**	**BC02**	AC24	Ssa07q^5^	Omy21q
40	BC02* and BC37	AC03p and AC24	Ssa17qb^5^	Omy15q
**10**	**BC03**	AC01q	Ssa12qa^2^	Omy17p
**10**	**BC03**	AC01p	Ssa12qb	Omy17q
**21**	**BC04**	AC13p	Ssa23	Omy04p
**38**	**BC04**	AC13q	Ssa04p^4^	Omy10q
**16**	**BC05**	AC27p	Ssa16qb^7^	
**16**	**BC05**	AC27q	Ssa29	Omy15p
**2**	**BC06**	AC15p	Ssa24	Omy06p
**2**	**BC06**	AC10	Ssa26^6^	Omy06q
**18**	**BC07**	AC06q	Ssa05p	Omy14p
**18**	**BC07**	AC06p	Ssa05q^1^	Omy14q
**1**	**BC08**	AC21	Ssa13qb	Omy12q
**1**	**BC08**	AC20b	Ssa03q^3^	Omy12p
7	BC09	AC23	Ssa04q	Omy10p
26	BC10	AC05	Ssa11qb	Omy sex
24	BC11	AC19	Ssa03p	Omy28
5	BC12	AC01 and AC40	Ssa01p	Omy19q
22	BC13	AC29	Ssa01qb	Omy05p
1	BC14	AC20a	Ssa06p^3^	Omy13q
17	BC15	AC04q	Ssa10qb	Omy02q
17 and 27	BC15 and BC17	AC16 and AC04q	Ssa10qa	Omy05q
	BC16		Ssa19qa	
23	BC18	AC17qa	Ssa13qa	Omy16q
15	BC19	AC28	Ssa15qa	Omy08p
20	BC20	AC26	Ssa16qa	Omy01p
12	BC21	AC11	Ssa22	Omy07q
8	BC22	AC32	Ssa14qa	Omy08q
9	BC23	AC31	Ssa27	Omy18q
32	BC24	AC02	Ssa25	Omy03q
14	BC25	AC22	Ssa20qb	Omy27
6	BC26		Ssa21	Omy22
	BC27	AC08p	Ssa28	
30 and 39	BC28	AC10	Ssa11qa^6^	Omy26
25	BC29	AC35	Ssa02p^1^	Omy03p
13	BC30	AC07	Ssa15qb	Omy09q
19 and 31	BC31	AC14q	Ssa06q	Omy04q
17	BC32	AC37	Ssa18qb	
35 and 36	BC33	AC08q	Ssa09qb	Omy20p
28	BC34	AC30	Ssa14qb	Omy14p
3	BC35	AC04q	Ssa09qa	Omy25
33	BC36	AC25	Ssa18qa	Omy01q
11	BC38	AC04p	Ssa09qc	Omy24
16*	BC39	AC12	Ssa17qa^7^	Omy07p
15	BC40	AC33	Ssa20qa	Omy11q
36	BC41	AC13q and AC34	Ssa08q^4^	Omy19p
	BC42	AC01q	Ssa02q^2^	

Atlantic Salmon chromosome arms annotated with numbers 1–8 in superscript represent arms with evidence for residual tetrasomy as identified in Lien *et al.* (2011, 2016). Brook Trout linkage groups in bold represent metacentric chromosomes.

### Comparisons with Rainbow Trout and Atlantic Salmon

Comparisons with the Atlantic Salmon genome found synteny for all 50 chromosomal arms. However, there were two incidences of two Brook Trout linkage groups [from Sutherland *et al.* (2016)] matching the same Atlantic Salmon chromosome arm: BC02 and BC37 both matched Ssa17qb (although note that support for a match between BC02 and Ssa17qb was weak) and BC15 and BC17 matched Ssa10qa. See Oxford plots showing synteny in File S4.

A total of 613 Brook Trout loci mapped to unique positions on the draft *O. mykiss* genome (Berthelot *et al.* 2014). A total of 40 Brook Trout linkage groups could be aligned to the Rainbow Trout genome (BC16 and BC27 did not align). One-to-one orthology could be confirmed for 11 chromosomes, including five retained metacentric chromosomes between Brook Trout and Rainbow Trout (BC02, BC03, BC06, BC07, and BC08, which match both arms of Omy21, Omy17, Omy06, Omy14, and Omy12, respectively) and six acrocentric chromosomes (BC26, BC38, BC35, BC28, BC25, and BC11, which match Omy22, Omy24, Omy25, Omy26, Omy27, and Omy28, respectively). Four Rainbow Trout chromosome arms matched two Brook Trout linkage groups (Omy01p, Omy02q, Omy13q, and Omy14p). One *O. mykiss* chromosome (Omy04) matched three Brook Trout linkage groups (LG19, LG21, and LG31: See Table 2). Six rainbow Trout chromosome arms (Omy02p, Omy09p, Omy11p, Omy13p, Omy18p, and Omy20q) could not be matched to the Brook Trout linkage map. See Oxford plots showing synteny in File S4.

## Discussion

The development of RADseq methodology has increased the amount of genetic information available for nonmodel organisms, including *Salvelinus*. Linkage maps that were once limited to several hundred microsatellite markers (Timusk *et al.* 2011; Sauvage *et al.* 2012) can now be constructed using many thousands of SNP loci (Sutherland *et al.* 2016; Nugent *et al.* 2017; study herein). Despite this increase in data, making comparisons between different linkage maps is complicated by a lack of shared markers between maps. This limitation has led to the development of software, such as MapComp (Sutherland *et al.* 2016), which allows comparisons to be made between different linkage maps by mapping loci to a related genome. Here, we use MapComp to compare homology between the map presented herein and linkage maps from Brook Trout (Sutherland *et al.* 2016), Arctic Char (Nugent *et al.* 2017), Rainbow Trout (Palti *et al.* 2015; Miller *et al.* 2012), and Atlantic Salmon (Lien *et al.* 2011, 2016) using the Atlantic Salmon genome as a reference. These results contribute to our understanding of genome organization within *Salvelinus*, between *Salvelinus* and *Salmo*, and between *Salvelinus* and *Oncorhynchus*.

### Comparisons within Salvelinus

The linkage map consists of 42 linkage groups, which corresponds with the diploid number of chromosomes in this species (2n = 84; Phillips and Rab 2001). Comparisons with Sutherland *et al.* (2016) confirm the presence of eight metacentric chromosomes (BC01–BC08; Table 2). However, there were some differences in the structure of the metacentric chromosomes between the Brook Trout maps. For example, BC04 matched two linkage groups (21 and 38) in the present map instead of just one linkage group as reported in Sutherland *et al.* (2016). This most likely represents oversplitting of one linkage group rather than karyotype variation between populations of Brook Trout in the structure of BC04. Additional evidence for variation in metacentric chromosomal arrangements includes a linkage group in the current study that matched both BC02 and BC37 in Sutherland *et al.* (2016). This linkage group also matched both AC03q and AC24 in Arctic Char (Nugent *et al.* 2017), suggesting merging of two separately inherited chromosome arms in the study herein. However, comparisons with the Atlantic Salmon genome found homology between the two Atlantic Salmon arms (Ssa17qb and Ssa07q) that matched this linkage group in the study herein. These two arms are known to form tetrasomic pairing in Atlantic Salmon during meiosis. The conservation of this tetrasomic pairing between *Salvelinus* and *Salmo* suggests possible residual tetrasomy between BC02 and BC37, rather than differences in chromosome arrangement.

Homology could be confirmed for 31 out of 34 acrocentric chromosomes when compared to Sutherland *et al.* (2016), with BC27, BC42, and BC37 failing to produce alignments with linkage groups in the study herein. This is almost certainly due to the low number of mapped markers rather than differences in the organization of the genome between the two mapping populations. There were three incidences of two linkage groups in the study herein matching one linkage group in Sutherland *et al.* (2016) (BC28, BC31, and BC33). Again, these most likely resulted from oversplitting of linkage groups, rather than differences in the karyotype between different populations of Brook Trout.

Comparisons were also made between Brook Trout and Arctic Char to determine variation in karyotype between two species of *Salvelinus*. The Arctic Char genome contains nine metacentric chromosomes (AC01, AC03, AC06, AC08, AC13, AC14, AC15, AC18, and AC27), of which five (AC01, AC06, AC13, AC18, and AC27) are homologous with metacentric chromosomes in Brook Trout (BC03, BC07, BC04, BC01, and BC05, respectively), suggesting conservation of these metacentric chromosomes among *Salvelinus* (Table 2). Two of the remaining four metacentric chromosomes in Arctic Char show homology with one arm of a metacentric chromosome in Brook Trout (AC03p and AC15p, which match BC02a and BC06a, respectively). The missing arms from both these Arctic Char metacentric chromosomes did not match any Brook Trout linkage groups, suggesting either fusion events that are specific to Arctic Char or inaccuracies in our comparative mapping approach. Nugent *et al.* (2017) report that AC03q and AC15q are homologous with Atlantic Salmon Ssa07q and Ssa26, respectively. As Ssa07q and Ssa26 are homologous to BC02b and BC06b, respectively, we believe that AC03 and AC15 are homologous with BC02 and BC06 and that the lack of homology between the q arms of these Arctic Char chromosomes and Brook Trout are due either to inaccuracies or (more likely) a low number of mapped markers in these regions. The remaining two Arctic Char metacentric chromosomes (AC08 and AC14) each match multiple Brook Trout linkage groups (BC27 and BC33, and BC31 and BC16, respectively) suggesting that these fusions are not shared by Brook Trout. Further linkage maps constructed from other species of *Salvelinus* will determine if the metacentric organization of these chromosomes is specific to Arctic Char, of if four acrocentric chromosomes represent fissions that are unique to Brook Trout.

The Arctic Char karyotype contains two split metacentric chromosomes, AC04 and AC20. Arctic Char vary in their structure of ACO4, where individuals can have either one metacentric chromosome (type 1) or one metacentric and one acrocentric chromosome (type 2). The population used by Nugent *et al.* (2017) is type 2 and comparisons with Brook Trout confirm homology of AC04p to BC38 and AC04q to BC35 and BC15, suggesting either a fusion event that is unique to Arctic Char or a fission event that is unique to Brook Trout. Comparisons to the Atlantic Salmon genome for BC15, BC35, and BC38 matched Ssa10qb, Ssa09qa, and Ssa09qc, respectively. As none of these chromosome arms represent known homeologies, and this split metacentric chromosome comprises three acrocentric chromosomes in Brook Trout, it is likely that this fusion is unique to Arctic Char. Similarly, comparisons to Brook Trout suggest that AC20 matches both BC08 and BC14. Comparisons to the Atlantic Salmon genome also found that both arms of AC20 matched with chromosome arms that are not homeologous (Ssa06p and Ssa03q), confirming that this metacentric chromosome is unique to Arctic Char.

### Evidence of homeologous relationships in Brook Trout

The genome duplication event that occurred in the ancestors of the modern-day salmonids resulted in a tetrasomic genome that is in the process of returning to a diploid state. However, several regions of the genome are still undifferentiated and form tetrasomic pairings in meiosis (Allendorf and Thorgaard 1984; Berthelot *et al.* 2014; Lien *et al.* 2016). Chromosomal arm homologies were inferred between Brook Trout and Atlantic Salmon, Rainbow Trout, and Arctic Char by comparative mapping approaches (see *Materials and Methods*). These homologies were used to identify potential tetrasomic inheritance patterns in Brook Trout (Table 3). Homeologous relationships seem to be conserved within *Oncorhynchus* (Kodama *et al.* 2014), between *Oncorhynchus* and *Salmo* (Lien *et al.* 2016), and between *Salvelinus*, *Oncorhynchus*, and *Salmo* (Nugent *et al.* 2017). Seven of the eight metacentric Brook Trout chromosomes contain one arm exhibiting residual tetrasomy in Atlantic Salmon (Ssa05q matched BC07, Ssa12qa matched BC03, Ssa03q matched BC08, Ssa04p matched BC04, Ssa07q matched BC02, Ssa26 matched BC06, and Ssa16qb matched BC05, respectively), suggesting that metacentric chromosomes in Brook Trout resulted from a tetrasomically-inherited arm fusing with an acrocentric chromosome that has diplodized; similar results were found in Arctic Char (Nugent *et al.* 2017). The chromosome organization of Atlantic Salmon is unusual in that Atlantic Salmon have seven rather than eight tetrasomic homeologous pairs, as found in *Oncorhynchus* (Kodama *et al.* 2014; Lien *et al.* 2016). Our comparative mapping approach with Rainbow Trout suggests an additional tetrasomic pairing involving BC01b and BC36. BC01b and BC36 match Omy23 and Omy01q, respectively, and additional evidence for tetrasomy was found for the two linkage groups that matched these chromosome arms in Arctic Char (AC18q and AC25, respectively). Evidence for this tetrasomic pairing in *Salvelinus* is especially compelling, as it has been proposed that one of the two chromosome arms that makes up a tetrasomic pairing needs to be from a metacentric chromosome (Kodama *et al.* 2014). However, evidence from Lien *et al.* (2016) suggests that fused acrocentric chromosomes can also provide the structure necessary for homeologous recombination.

**Table 3:** Homeologous chromosome pairs in Brook Trout after the salmonid-specific whole genome duplication event [as determined by linkage maps in study herein and Sutherland *et al.* (2016)]

Homeolog 1	Homeolog 2
BC01a	BC05b
BC01b*^a^*	BC36*^a^*
BC02a^5^	BC37^5^/BC02b*^b^*
BC02b	BC37/BC02a*^b^*
BC03a^2^	BC42^2^
BC03b	BC21
BC04a	BC17
BC04b^4^	BC41^4^
BC05a^7^	BC39^7^
BC06a	BC40
BC06b^6^	BC28^6^
BC07a	BC33
BC07b^1^	BC29^1^
BC08a	BC09
BC08b^3^	BC14^3^
BC10	BC13
BC11	BC22
BC12	BC35
BC15	BC20
BC16	BC27
BC18	BC30
BC19	BC31
BC23	BC34
BC24	BC26
BC25	BC38
BC32	BC29

Current undifferentiated homeologous arms were identified through comparisons with the Arctic Char linkage map (Nugent *et al.* 2017) and the Atlantic Salmon genome (Lien *et al.* 2016). Chromosomes that still form tetrasomic pairings during meiosis are designated with superscript numbers 1–7. This numbering aids in comparisons between this table and Table 2 in Nugent *et al.* (2017). An additional chromosome pairing shows residual tetrasomy in *Oncorhynchus* (Kodama *et al.* 2014).

a Comparative mapping suggests a similar tetrasomic pairing may still occur in Brook Trout.

b Pairs with weak relationships.

### Comparisons with Atlantic Salmon

Comparison with Atlantic Salmon supported previous observations of genome evolution in salmonids. Chromosomes Ssa01 and Ssa09 in Atlantic Salmon were each formed through a fusion of three ancestral chromosome arms (Lien *et al.* 2016). Each of these chromosomes aligns to three linkage groups in our Brook Trout linkage map, and similar results have been found in other *Salvelinus* and *Oncorhynchus* linkage maps (Sutherland *et al.* 2016; Nugent *et al.* 2017; Kodama *et al.* 2014). These fusions in Atlantic Salmon arose after the lineages that gave rise to *Salvelinus* and *Oncorhynchus* split from *Salmo* during salmonid evolution. An additional five Atlantic Salmon chromosomes [Ssa06 (metacentric), Ssa10 (fused acrocentric), Ssa13 (fused acrocentric), Ssa14 (fused acrocentric), and Ssa20 (fused acrocentric)] each matched two Brook Trout linkage groups in the study herein. The same chromosomes also matched two linkage groups in Sutherland *et al.* (2016), further supporting multiple fusion events after *Salmo* split from the ancestor of *Oncorhynchus* and *Salvelinus*. Two metacentric Brook Trout linkage groups (BC01 and BC08) each matched two Atlantic Salmon chromosome arms (Ssa13qb and Ssa03q, and Ssa19qb and Ssa01qa, respectively). The alignment of linkage group BC01 to multiple Atlantic Salmon chromosomes appears to represent true karyotype differences between *Salvelinus* and Atlantic Salmon, as the same result was seen with AC18 in Arctic Char (Nugent *et al.* 2017). These consistent results suggest a fission event of the ancestral chromosome after *Salmo* split from the other salmonids. However, no such relationship was found with the Arctic Char chromosome arms that match BC08. Two other *Salvelinus*-specific fusions, BC04 (AC13) and BC05 (AC27), were not seen in other salmonids. Interestingly, both of these chromosomes are metacentric, suggesting that the fusion of these chromosomes happened after *Salvelinus* diverged from the other salmonids.

### Position of the sex marker in the Salvelinus genome

Our mapping results determined that the sex marker (*sdY*) mapped to BC35, in an area of reduced recombination in both males and females. This linkage group matched Ssa09qa in the Atlantic Salmon genome and AC04q in Arctic Char (Nugent *et al.* 2017). The location of *sdY* in salmonids has received a lot of interest, as *sdY* is part of a cassette that has moved to different chromosomes between species (Yano *et al.* 2012, 2013) and between different populations of Atlantic Salmon (Eisbrenner *et al.* 2014). The determination of sex via a cassette that jumps to different chromosomes is, so far, unique to the salmonids (Lubieniecki *et al.* 2015). Recently, Sutherland *et al.* (2017) also mapped sex to BC35, confirming its location in two populations of Brook Trout, suggesting that the sex causative locus has not moved to different chromosomes, at least in the Brook Trout populations studied. However, the same does not hold for other species of *Salvelinus*, as chromosome painting has determined that the sex marker has moved to different linkage groups in different populations of Arctic Char (AC04 in North American and AC01/21 in European Arctic Char; Phillips *et al.* 2006; Timusk *et al.* 2011). However, it must be stressed that the function of *sdY* in *Salvelinus* has not been determined. Follow-up studies that document patterns of gene expression in Brook Trout are necessary to determine if *sdY* is the master sex-determining gene in this species.

### Conclusions

Here, we present a linkage map for Brook Trout comprised of 42 linkage groups. Comparisons with other salmonid linkage maps confirmed some of the many fusion and fission events that have occurred both after *Salvelinus* and *Salmo* split from a common ancestor, and between Arctic Char and Brook Trout. Using comparative genomic approaches with software such as MapComp increased our understanding of salmonid genome evolution, particularly in chromosome arms that are undifferentiated and can exhibit tetrasomic inheritance.

## Supplementary Material

Supplemental material is available online at www.g3journal.org/lookup/suppl/doi:10.1534/g3.117.300317/-/DC1.
