# Supplementary material for: Using Linkage Maps as a Tool To Determine Patterns of Chromosome Synteny in the Genus Salvelinus
Source: G3 (Bethesda). 2017 Sep 29;7(11):3821–30. doi: 10.1534/g3.117.300317 (PMC5677171; doi:10.1534/g3.117.300317)

Supplemental material 4A: Oxford grid comparing Rainbow trout linkage groups in Miller et al. (2012) to linkage groups published herein. MAPCOMP was used for inferring homology. The Atlantic salmon genome was used to map RADtags.

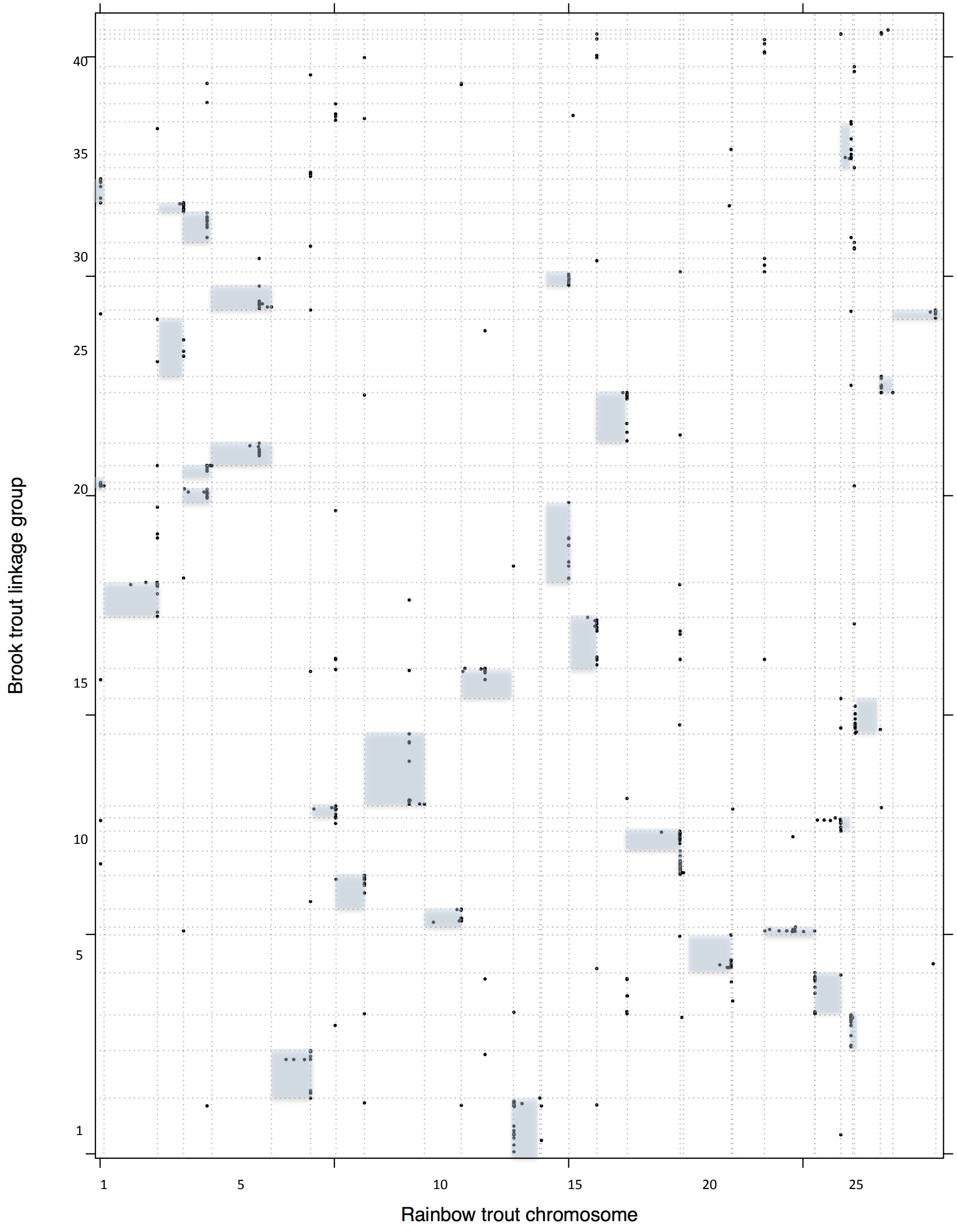

Supplemental material 4B: Oxford grid comparing Brook trout linkage groups to the Atlantic salmon genome (distance in MBs).

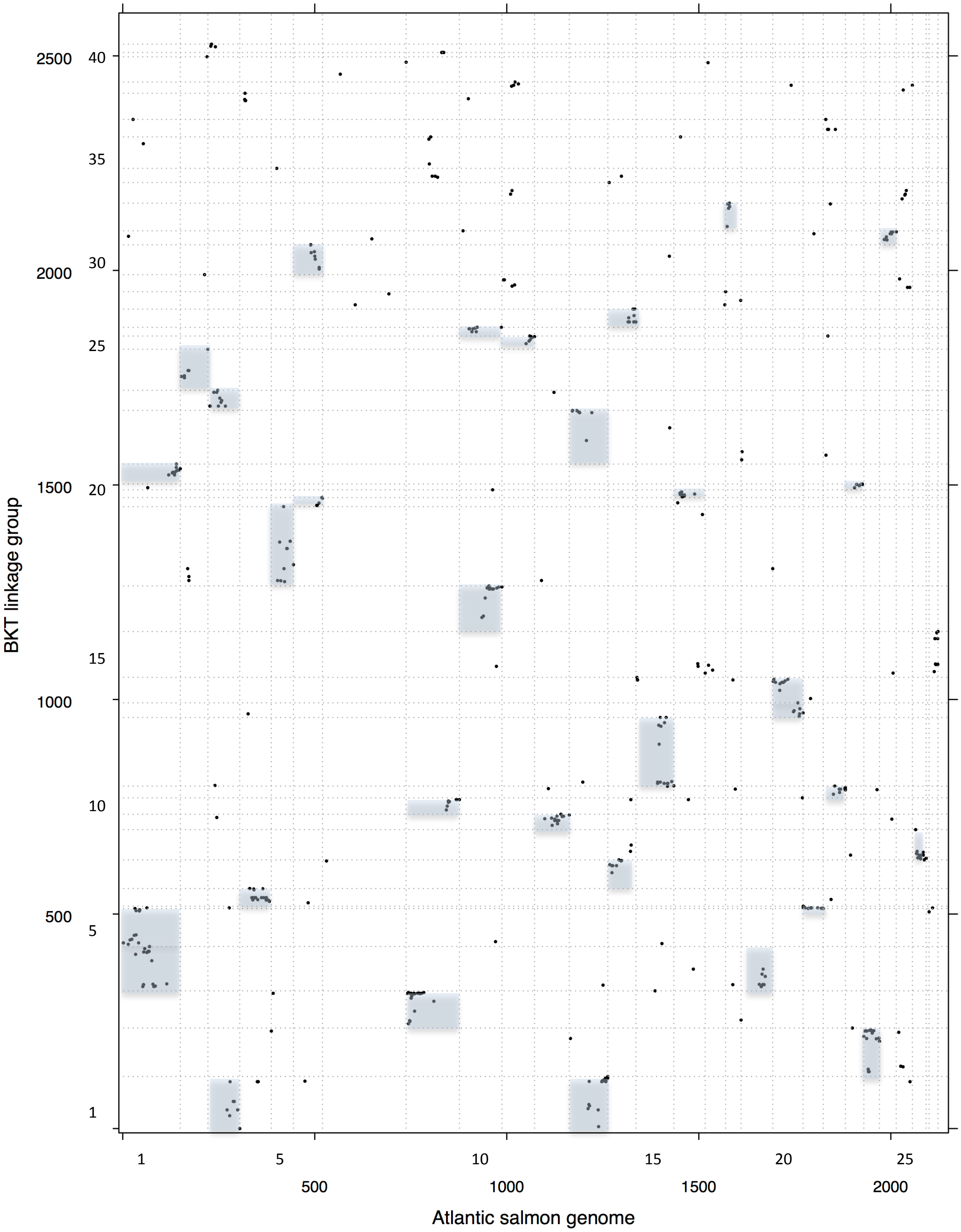

Supplemental material 4C: Homologous linkage groups between Hale et al and Sutherland et al (2016).

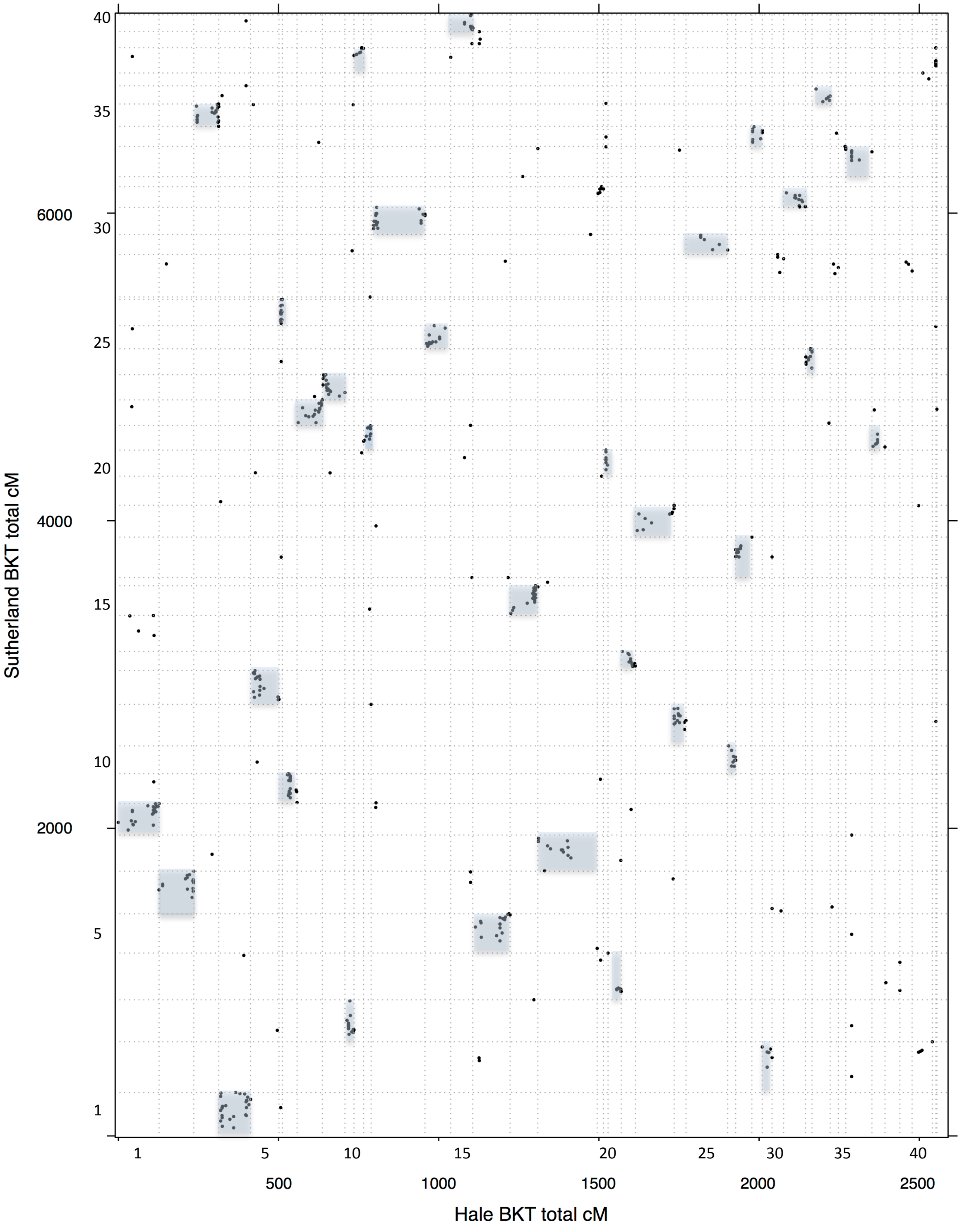

Supplemental material 4D: Oxford plots showing homologous relationships between the current study and Arctic char (Nugent et al. 2017).

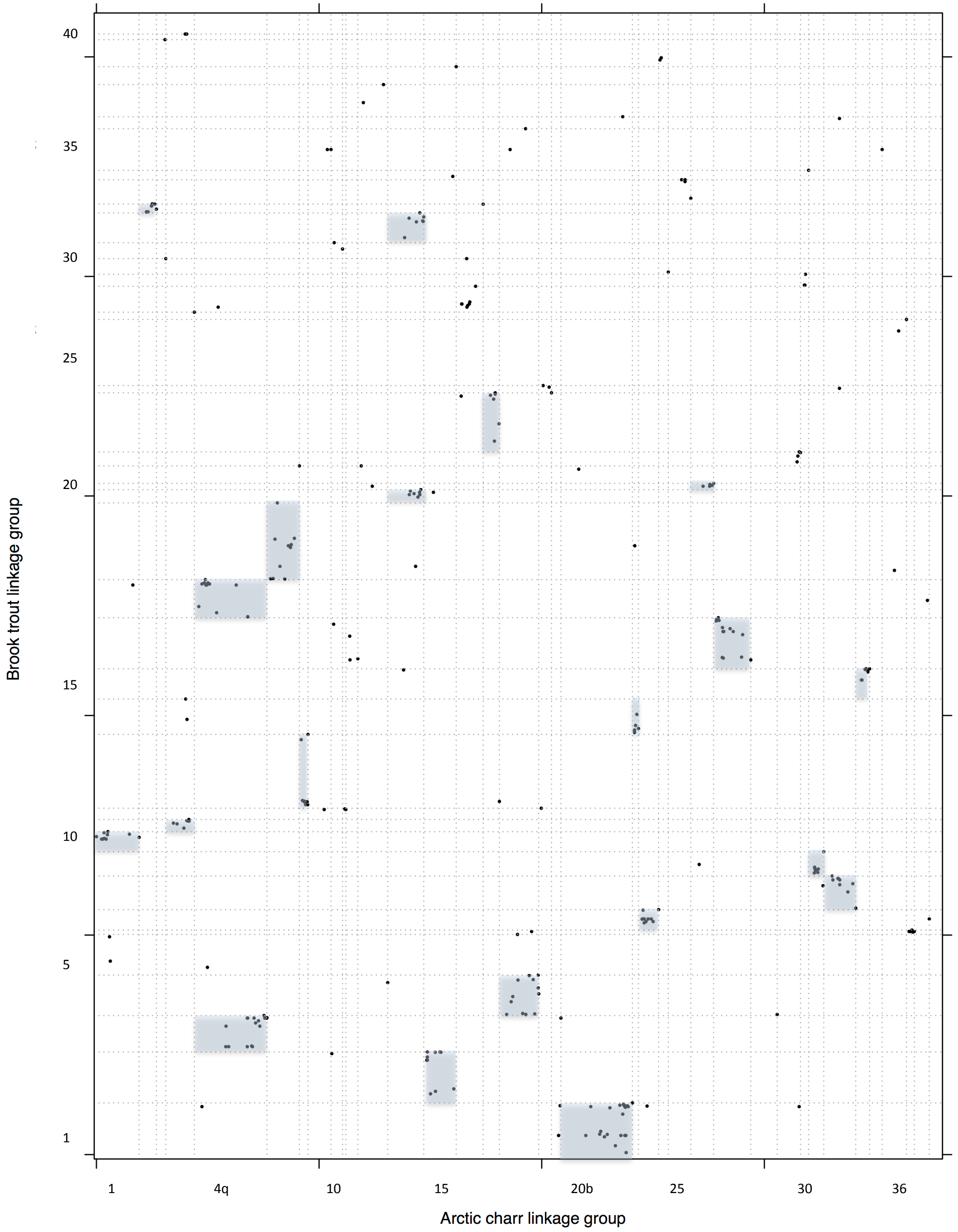

Supplement: Supplementary file 4 [file 3821FileS4.pdf]
